# The discrepancies in parent and teacher reports of children’s behavioral inhibition provide domain-specific information about psychopathology and parenting

**DOI:** 10.3389/fpsyt.2024.1457479

**Published:** 2024-11-25

**Authors:** Róza Sára Sulyok, Mónika Miklósi, Noémi Kárpáti, Szandra Györe, Brigitta Szabó

**Affiliations:** ^1^ Doctoral School of Psychology, ELTE, Eötvös Loránd University, Budapest, Hungary; ^2^ Department of Clinical Psychology, Faculty of Medicine, Semmelweis University, Budapest, Hungary; ^3^ Institute of Psychology, Eötvös Loránd University, Budapest, Hungary; ^4^ Centre of Mental Health, Heim Pál National Pediatric Institute, Budapest, Hungary

**Keywords:** behavioral inhibition, preschool, parent-report, teacher-report, informant discrepancies, parenting, internalizing problems, externalizing problems

## Abstract

**Introduction:**

Behavioral inhibition is a temperamental factor that increases the risk of internalizing disorders. Therefore, the identification of highly inhibited children is of great importance. However, informant discrepancies make this process difficult. In a cluster analytic approach, we aimed to use both parent and teacher reports of behavioral inhibition in order to gain a more detailed picture about children’s behavioral inhibition in different contexts and to characterize highly inhibited children.

**Methods:**

Parents and teachers of 318 preschool children completed a questionnaire, which included the Behavioral Inhibition Questionnaire (BIQ) and the Strengths and Difficulties Questionnaire (SDQ). Parents also reported their parenting behavior on the Multidimensional Assessment of Parenting Questionnaire (MAPS). A two-step cluster analysis was conducted on BIQ parent and teacher reports, and the resulting clusters were compared on the SDQ externalizing and internalizing subscales. Multinomial logistic regression analyses were conducted separately for girls and boys to predict cluster membership based on the MAPS hostility, lax control and physical control subscales.

**Results:**

Four clusters were identified, labelled as medium-low (ML), low-elevated (LE), elevated-elevated (EE) and high-high (HH), based on the levels of BIQ parent and teacher reports, respectively. In the HH cluster, mean scores of the SDQ internalizing subscales as reported by parents and teachers were significantly higher, and in boys but not in girls, mean scores of the SDQ externalizing subscale as reported by teachers were lower than in the other clusters. High levels of hostility predicted group membership of HH compared to LE and EE in both genders. Furthermore, in boys, lax control and physical control were also found to be significant when comparing HH to EE and LE, respectively.

**Discussion:**

Our results suggest that the joint use of parent and teacher reports on behavioral inhibition may increase the ability to identify highly inhibited children at risk of developing internalizing disorders and add to our understanding of the underpinnings of children’s inhibited behavior in different contexts.

## 1 Introduction

In the last few decades, researchers examined childhood shyness from many aspects. In 1984, Kagan and colleagues (1) introduced a novel theoretical framework that posited a temperamental characteristic underlying childhood shyness, termed behavioral inhibition. Behavioral inhibition is an early-appearing, biologically determined trait (2) with a prevalence of 10-15% (1) or even 15-20% (3). Children with high levels of behavioral inhibition demonstrate withdrawal and negative emotions in the face of new social or non-social situations. This reaction is related to a hyperactive physiological stress system and is typically associated with specific physiological responses such as increased heart rate, dilated pupils, and higher cortisol levels (2, 4–6). As a result, highly inhibited children frequently demonstrate a reluctance to explore unfamiliar individuals, locations, and objects, and to refrain from gaining familiarity with them. Additionally, they tend to respond with caution, hesitation, apprehension, or avoidance (6, 7).

Behavioral inhibition is regarded as an early risk factor for internalizing disorders, including depression and anxiety disorders, particularly social anxiety disorder (2, 8–11). Therefore, detecting behavioral inhibition at an early age to reduce the risk of psychopathology is warranted (11, 12).

Though behavioral inhibition is a relatively stable trait (13), environmental factors may affect its developmental course and moderate its association with internalizing disorders. One major environmental factor that interacts with early temperament such as behavioral inhibition to significantly shape developmental trajectories is parenting (2). A growing body of evidence suggests that overprotective and overly sensitive parenting may be associated with greater stability in behavioral inhibition and more anxious behavior (14–19). These parenting behaviors serve to maintain behavioral inhibition, as they prevent the child from gaining the opportunity to explore new environments (2). Negative parenting behaviors, including intrusiveness, overcontrol, low autonomy granting, low support and criticism have similar effect in maintaining behavioral inhibition and increasing the risk of anxiety (15, 20, 21). These parenting behaviors may result in overwhelming the child in new situations, thereby undermining the child’s self-regulating ability (2). However, some of these associations may be bidirectional, indicating an evocative effect of behavioral inhibition on parenting (14) (15, 20, 22, 23).

Parental behavior is a modifiable factor and can therefore be the focus of early interventions (2). Thus, it is important to have an accurate picture of the relationship between behavioral inhibition and parental behavior.

Furthermore, gender may be a significant factor in the relationship between parental behavior and behavioral inhibition (6). Although no differences in the prevalence of behavioral inhibition have been found in early childhood (24), several researches have shown that the developmental trajectories and psychopathology may differ by gender in behavioral inhibition (6, 25, 26). The observed developmental differences, in addition to biological factors, may be explained by socialization factors, such as the greater acceptance of inhibited behavior in women (27, 28). Several studies have demonstrated that the associations between children’s behavior and parental behavior may differ depending on the gender of the child. However, the differences are not yet clear and may depend on the degree of behavioral inhibition (17, 25, 29).

In evaluating behavioral inhibition, the source of the report is of significant importance. Clinical observation is a rarely used method, primarily due to its high cost, time-consuming nature, and the potential for information to be distorted or lost in artificial settings (4, 30). The most common method of assessing the development and behavior of young children in the preschool years is through parent or teacher reports, or a combination of both (31). However, previous studies have shown that teacher and parental evaluations of children’s behavioral inhibition have a low to moderate correlation (7, 12, 32). Furthermore, the correlates of behavioral inhibition may differ across informants and contexts. In a recent study conducted by Espinoza-Fernandez and colleagues (33), the researchers collected data on children’s behavioral inhibition from two distinct contexts: the family context, which included data from mothers and fathers, and the school setting, which included data from teachers. The findings indicated that children with behavioral inhibition could be characterized by high levels of shyness regardless of informants; however, teachers but not parents reported higher levels of somatic complaints but did not score lower social and adaptive skills in their identified behaviorally inhibited students.

The occurrence of informant discrepancies has been identified as a highly consistent finding in the field of children’s mental health research. In both older and more recent meta-analyses, an overall correspondence of 0.28 appears to be a relatively stable finding over time (34, 35), countries (36), and across the life-span (37). Furthermore, this correlation was higher for the externalizing than the internalizing domain (31).

Early theories posited that informant discrepancies were attributable to measurement error (see (38), for a review). Consequently, their efforts were directed at capturing the common variance of data from different informants. However, recent research demonstrated that informant discrepancies frequently reflect domain-relevant information, that enhances our comprehension of the phenomena under investigation (39). For example, specific patterns of informant discrepancies have been shown to predict poor outcomes in internalizing disorders (40), suicidal thoughts in adolescent depression (41), and educational outcomes in autism spectrum disorder (42). Furthermore, patterns of discrepancies between mothers’, fathers’ and teachers’ reports on limited prosocial emotions have been identified as a risk factor for conduct problems in a clinical sample of elementary school children (43).

Consequently, three interrelated theoretical models have been developed with the objective of demonstrating new ways of addressing information discrepancies in clinical and research settings.

In order to facilitate research, the Operations Triad Model (OTM) (44) has been proposed as a framework for conceptualizing the patterns of data observed within multi-informant assessment. Previous research methodology has been largely based on the concept of *converging operations*, which implies the view that convergence from multiple methodologies lends support to the validity of the results, whereas discrepancies indicate the potential presence of some forms of error. In contrast, the OTM model posits that domain-relevant variance may originate from both converging and diverging reports (*divergent operations*). However, this must be distinguished from discrepancies that are attributable to measurement confounds (*compensating operations*) (39).

In clinical settings, the Attribution Bias Context Model (ABC Model) has been put forth as a prospective framework for interpreting informant discrepancies (45). The model proposes that discrepancies between sources of information may be attributed to three factors and their interactions. The initial factor is the cause attributed to the child’s problem, which is also known as the actor-observer phenomenon. For instance, a conflict may be perceived in different ways, with the cause attributed to either an intrinsic factor, such as the child’s inherent propensity for aggression, or to an extrinsic factor, such as the child being placed in a challenging situation with no viable alternative. The second factor is the informant’s perspective, which may influence how memories are recalled. Informants may hold disparate opinions regarding the perceived most significant issue in the context in which they observe the child, as well as the necessary changes they believe are required. According to the ABC Model, respondents will recall memories and report symptoms in a manner that reflects their perspectives. Finally, the extent to which the respondents agree with the objectives of the assessment, for instance, whether or not they would like a diagnosis or treatment for the child, may also influence their responses. These factors may interact to determine which memories respondents evoke when assessing the child’s behavior and how they report them.

Interpreting discrepancies among information sources can play a significant role in clinical decision-making, particularly in defining needs and goals. To address this issue, the Needs-to-Goals Gap Framework (46) was developed based on the ABC and OTM models. In this model, a decision is made regarding the reliance on one or multiple informants in the need assessment phase, and the parallel and/or independent functional assessment across contexts in the goal phase, according to the consistency of the reports about the needs across contexts. This would reduce the risk of a needs-to-goals gap in service delivery, whereby the goals set for service delivery fail to align with the needs of the client (39).

The necessity for an explanation of informant discrepancies also emerged in the field of behavioral inhibition research. It has been postulated that children are assumed to feel safer in the presence of a parent, which has led to the prediction that parental reports typically indicate lower behavioral inhibition than teacher reports (7). Ballespí and colleagues (12) conducted a study to analyze parent-teacher discrepancies in reporting children’s behavioral inhibition. Results revealed that the correlation between parent and teacher reports is higher in the case of social than non-social signs of behavioral inhibition. Furthermore, the agreement was also higher on items related to speech and avoidance behavior and shyness with adults than for other behaviors. In accordance with the ABC Model (45), they suggested that the higher correlation reflects the highest importance of these behaviors for both types of informants (12). Moreover, both parents and teachers were found to have a moderate-to-low ability to identify behaviorally inhibited children (12). Consequently, the authors underscored the necessity to view parental and teacher reports as supplementary rather than equivalent. Nevertheless, no research has yet attempted to address this question. Such research, which employs parent and teacher reports as a complementary data source, would presumably be more successful in identifying highly inhibited children. This approach could then be utilized to investigate the risk and protective factors, including parenting, that shape developmental trajectories of behavioral inhibition and increase or decrease the subsequent risk of psychopathology.

A person-centered approach may prove advantageous over traditional variable-centered approaches when exploring patterns of informant discrepancies (47). For instance, cluster analysis can be employed to delineate emerging subgroups based on both informant severity ratings and the degree of informant discrepancy (48). These subgroups can then be explored in psychopathology and parental variables. Therefore, the objective of this study was to employ a cluster analytic approach to utilize both parent and teacher reports of behavioral inhibition in a sample of preschool children.

In accordance with the Operations Triad Model (44), it was hypothesized that discrepancies between informants in ratings of children’s behavioral inhibition, as reported by parents and teachers, reflect domain-relevant information that is clinically meaningful. We expected that differences in parental and teachers’ assessments of behavioral inhibition severity would yield distinct subgroups within the population, which could be uncovered by cluster analysis.

Specifically, we postulated that agreement between parental and teacher reports on high behavioral inhibition would more accurately identify the group at risk for anxiety disorders, than severity ratings. Consequently, we anticipated that this group would exhibit higher scores on the SDQ internalizing scale based on both parent and teacher reports than the other groups (i.e. disconcordant parental and teacher reports).

We supposed that this approach would also help to gain a more accurate picture of the relationship between high levels of behavioral inhibition and parental behavior. More specifically, we explored the association between agreement on high behavioral inhibition between parents and teachers and three negative parenting behaviors assessed by the Multidimensional Assessment of Parenting Scale (49), hostility, which includes criticism, harshness and intrusiveness, lax control, which is characterized by permissiveness and undercontrol, and physical punishment.

## 2 Materials and methods

### 2.1 Sample and procedures

The Institutional Research Ethics Committee of the Psychological Institute Eötvös Loránd University approved the study (Nr. 2019/250). The participants were recruited through preschools. After giving their written informed consent, the parents received a questionnaire packet which they filled in at home and returned to the investigator in a sealed envelope. Teachers completed the questionnaire for each child whose parents had consented to the study and returned it in a sealed envelope to the investigator.

The data of 318 caregivers of preschool-aged children were analyzed (298 mothers, 12 fathers and 8 other caregivers). The mean age of the respondents was 36.02 years (*SD* = 6.47, range: 20  –64 years). The highest level of education was low (≦ 8 years of education) for 23 (7.2%), medium (12 years) for 158 (49.7%), and high (>12 years) for 137 (43.1%) caregivers. Place of residence was the capital for 130 (40.9%) respondents, 164 (51.6%) of them lived in urban, and 24 (7.5%) in rural areas. The mean age of the children was 4.61 years (*SD* = 0.97, range: 3 - 7 years), and there were 165 (51.9%) boys and 153 (48.1%) girls among them.

### 2.2 Measure

The Behavioral Inhibition Questionnaire [BIQ (7, 50)] was employed to assess behavioral inhibition. This widely used questionnaire is designed to assess behavioral inhibition in both social and non-social contexts. It is available in two versions: the parent report form comprises 30 Likert-type items, while the teacher report version contains 28 items of the same type. The items pertain to various situations, including interactions with unfamiliar adults, peers, physical challenges, toys, separation and performance. The parental version incorporates two supplementary items concerning the behavior of children in unfamiliar domestic settings. Although behavioral inhibition may manifest in various forms across novel social and situational contexts, it was deemed redundant to examine these variables separately in the present study due to their high correlation. Therefore, a total score was used. Although the Behavioral Inhibition Questionnaire lacks a validated cut-off, studies have employed two distinct methodologies for identifying children who exhibited behavioral inhibition. One approach involves establishing a percentage limit based on the top 15-20% scores (7). An alternative approach is to achieve a score of 132 or above (7, 51, 52). However, neither method was employed in the course of our analyses. Instead, cluster analysis was conducted using both parent and teacher reports in order to identify subgroup of children exhibiting high levels of inhibition. In our data, both the parent and the teacher report versions demonstrated excellent internal consistency (**Table 1**).

**Table 1:** Cluster characteristics, descriptive statistics and reliabilities of study variables.

Cluster	ML (medium-low)	LE (low-elevated)	EE (elevated-elevated)	HH (high-high)	Total	α
Boys (%)	43.8	55.9	52.9	57.6	51.9	NA
Mean age in years (*SD*)	4.64 (1.08)	4.63 (0.83)	4.64 (0.92)	4.50 (1.01)	4.61 (0.97)	NA
BIQ parent report						0.94
mean (*SD*)	82.26 (19.44)	60.37 (10.41)	103.19 (14.00)	137.01 (22.34)	96.41 (30.77)	
range	34 – 134	40 – 78	80 – 134	91 – 206	34 – 206	
scores ≥ 132, *N* (%)	1 (1.1)	0 (0)	1 (1.0)	41 (62.1)	43 (13.5)	
BIQ teacher report						0.95
mean (*SD*)	64.69 (15.19)	105.48 (16.32)	108.15 (13.22)	135.47 (23.36)	101.16 (30.32)	
range	28 – 90	77 – 147	78 – 141	80 – 178	28 – 178	
scores ≥ 132, *N* (%)	0 (0)	3 (5.1)	3 (2.9)	36 (54.5)	42 (13.2)	
SDQ-I parent report, mean (*SD*)	2.37 (2.10)	1.80 (1.44)	3.24 (2.35)	5.45 (2.83)	3.18 (2.57)	0.68
SDQ-I teacher report, mean (*SD*)	1.82 (2.16)	4.62 (2.85)	4.42 (2.90)	6.51 (3.74)	4.18 (3.33)	0.77
SDQ-E parent report, mean (*SD*)	7.23 (4.11)	6.57 (3.80)	6.38 (3.79)	7.40 (4.08)	6.86 (3.94)	0.82
SDQ-E teacher report, mean (*SD*)	6.37 (4.75)	7.09 (4.92)	5.41 (4.29)	3.91 (3.97)	5.69 (4.59)	0.88
MAPS-LC, mean (*SD*)	2.03 (0.64)	1.79 (0.58)	2.06 (0.62)	1.85 (0.49)	1.96 (0.60)	0.79
MAPS-HS, mean (*SD*)	2.35 (0.70)	2.01 (0.52)	2.10 (0.55)	2.31 (0.63)	2.20 (0.62)	0.83
MAPS-PC, ‘no use’ *N* (%)	29 (32.6)	27 (45.8)	42 (40.4)	29 (43.9)	127 (39.9)	0.82
*N*	89	59	104	66	318	

BIQ, Behavioral Inhibition Questionnaire. The labels of the four clusters according to the parent-report and teacher-report BIQ scales, respectively, HH, high-high; EE, elevated-elevated; LE, low-elevated; ML, medium-low. SDQ-I, Strengths and Difficulties Questionnaire; Internalizing subscale. SDQ-E, Strengths and Difficulties Questionnaire; Externalizing subscale. MAPS-LC, Multidimensional Assessment of Parenting Scale; Lax Control subscale. MAPS-HS, Multidimensional Assessment of Parenting Scale; Hostility subscale. MAPS-PC, Multidimensional Assessment of Parenting Scale; Physical Control subscale (dummy-coded, 0 = no use; 1 = else). *SD*, standard deviation. NA, non-applicable.

The Strengths and Difficulties Questionnaire, parent and teacher report [SDQ (53, 54)] is a 25-item Likert-type scale (0: not true, 1: somewhat true, 2: certainly true) that assesses emotional and behavioral problems in children and adolescents. In this study, we employed the Internalizing Problems subscale (Emotional Problems and Peer Relationship Problems), and the Externalizing Problems subscale (Conduct Problems and Hyperactivity/Inattention), in accordance with the recommendations of Goodman and colleagues (55) for non-clinical samples. In our sample, the internal consistencies of the parent report version were found to be acceptable to good, while those of the teacher report version were found very good (**Table 1**).

The Multidimensional Assessment of Parenting Behavior (49) (Hungarian version (56)) was employed to assess parents’ perceptions of their parenting behavior. This questionnaire comprising 34 items, assesses positive and negative parenting behaviors on a five-point Likert scale. The psychometric properties have been demonstrated to be excellent by Parent and Forehand (49). Three subscales were employed in our study to assess negative parenting behaviors: hostility, lax control, and physical control. The internal consistencies of the subscales were found to be good in the present sample (**Table 1**).

### 2.3 Statistical analyses

Descriptive statistics and internal consistencies of the scales are reported. The association between parent and teacher reports was assessed by means of Pearson’s correlational coefficient. Gender differences were explored using independent t-tests.

A two-step cluster analysis was conducted in IBM SPSS Statistics (version 26) with two predictors, namely the parent and teacher report forms of the BIQ. The Silhouette measure of cluster cohesion was employed to assess the clustering quality. A score of > 0.5 indicates good clustering, a score between 0.5 and 0.25 indicates fair clustering, and a score of < 0.25 indicates poor clustering. The number of clusters was determined automatically using Schwarz’s Bayesian Criterion (BIC). The log-likelihood distance measure was used. The clusters were labelled according to the parent and teacher report BIQ levels, respectively. One-way ANOVAs were conducted to compare the mean scores of BIQ scales among the clusters. Tukey’s *post hoc* tests were used when the assumption of the equality of variances was met, and Dunnett’s C tests were used for *post-hoc* comparisons when the variances were unequal.

A series of two-way ANOVAs were conducted to analyze the effects of cluster membership and gender on parent and teacher report SDQ Internalizing and Externalizing subscales.

Finally, multinomial logistic regression analyses were conducted to explore the predictive power of the three negative parenting behaviors on cluster membership. Because of the previously reported differences in the relationships between behavioral inhibition and parenting by gender, we run the analyses separately in the samples of boys and girls.

In order to determine the appropriate sample size, it was considered that the expected sample size of the subgroups should be at least 20-30 (57) for both boys and girls. Given the 15-20% prevalence of high behavioral inhibition (8), the minimum sample size was set at 280. This is also larger than the sample size recommended for cluster analysis with two predictors (i.e. 2x70 = 140) (58).

## 3 Results

### 3.1 Descriptive statistics

**Table 1** presents the descriptive statistics and reliabilities of the scales. As nearly 40% of the parents indicated that they did not utilize physical control on the MAPS-PC subscale, we recoded it (0 = no use of PC, 1 = other) and used the dummy-coded scale for subsequent analyses (**Table 1**).

### 3.2 Preliminary analyses

The correlations of the BIQ social novelty and BIQ situational novelty subscales were 0.787 (*p* < 0.001) for the parent report, and 0.812 (*p* < 0.001) for the teacher report, therefore we used the total scores of the BIQ for the cluster analysis. The scores of the BIQ parent and teacher reports were significantly and positively related (*r* = 0.413, *p* < 0.001). There were no gender differences in BIQ parent report (*M_boys_* = 98.26, *SD_boys_* = 31.85, *M_girls_* = 94.41, *SD_girls_* = 29.53, *t*(316) = 1.115, *p* = 0.266, *d* = 0.126). However, boys’ mean scores were significantly higher than girls’ mean scores in the teacher report (*M_boys_* = 104.67, *SD_boys_* = 28.69, *M_girls_* = 97.38, *SD_girls_* = 31.65, *t*(316) = 2.155, *p* = 0.032, *d* = 0.241). The effect size was small.

### 3.3 Results of the two-step cluster analysis

The two-step cluster analysis revealed that a four-cluster classification was the optimal solution for the data. Predictor importance was 1 for both predictors. The average silhouette value of the model was 0.5. The largest cluster comprised 104 children (32.7%) and the smallest cluster consisted of 59 children (18.6%). The ratio of the cluster sizes (largest cluster to smallest cluster) was 1.76. The clusters were labelled as medium-low (ML), low-elevated (LE), elevated-elevated (EE), and high-high (HH) based on the parent and teacher report BIQ levels, respectively (**Table 1**).

One-way ANOVAs demonstrated that the clusters exhibited significant differences in parent and teacher report BIQ. When choosing the BIQ parent report as the dependent variable, the model was found to be significant (*F*(3,314) = 236.230, *p* < 0.001, η_p_^2^ = 0.693), and the Dunnett’s C *post-hoc* test revealed that all pairwise comparisons were significant (HH > EE > ML > LE). When the BIQ teacher report was used as the dependent variable, the model was also significant (*F*(3,314) = 237.240 *p* < 0.001, η_p_^2^ = 0.694). All but one pairwise comparison was significant (HH > EE = LE > ML).

There were no gender (χ^2^(3) = 3.604, *p* = 0.308), and age differences among clusters (*F*(3,314) = 0.360, *p* = 0.782) (**Table 1**).

### 3.4 The effect of cluster membership and gender on SDQ

**Figure 1:**
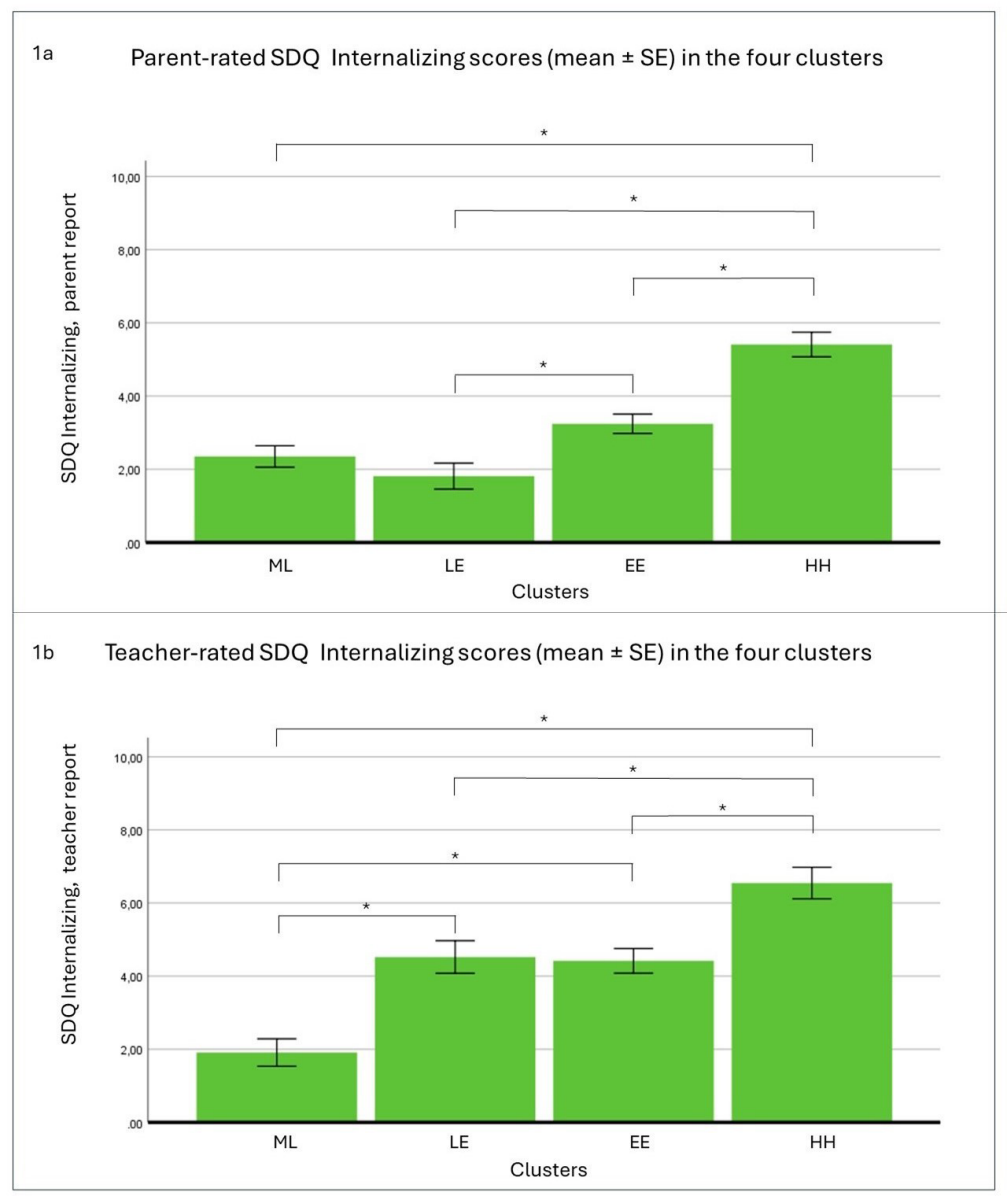
The effect of cluster membership on parent **(A)** and teacher **(B)** rated SDQ Internalizing scores. *N* = 228. SDQ: Strengths and Difficulties Questionnaire. The labels of the four clusters according to the parent-report and teacher-report BIQ scales: HH, high-hig; EE, elevated-elevated; ML, medium-low; LE, low-elevated; BIQ, Behavioral Inhibition Questionnaire; SE, standard error. * *p* < 0.05.

A two-way analysis of variance (ANOVA) was conducted to examine the influence of cluster membership and gender on parent-reported SDQ Internalizing subscale scores. The model was found to be significant (*F*(7, 227) = 10.389, *p* < 0.001, η_p_^2^ = 0.249). The results indicated that cluster membership had a statistically significant main effect on parent-rated SDQ Internalizing subscale scores (*F*(3, 227) = 22.270, *p* < 0.001, η_p_^2^ = 0.234). However, the main effect of gender was not significant (*F*(1, 227) = 0.092, *p* = 0.810, η_p_^2^ < 0.001). The analysis revealed no statistically significant interaction between the effects of cluster membership and gender (*F*(3, 227) = 0.321, *p* = 0.810, η_p_^2^ = 0.004). Tukey *post-hoc* analyses revealed that mean scores in the HH cluster were significantly higher than mean scores in the other three clusters, and the mean scores in the EE cluster were significantly higher than the mean scores in the LE cluster (**Table 1** and **Figure 1A**).

When choosing SDQ Internalizing, teacher reports, as the dependent variable, the analysis yielded a significant model (*F*(7, 227) = 11.125, *p* < 0.001, η_p_^2^ = 0.261). The main effect of cluster membership was significant (*F*(3, 228) = 22.562, *p* < 0.001, η_p_^2^ = 0.129), whereas the main effect of gender was not found to be significant (*F*(1, 228) = 2.324, *p* = 0.129, η_p_^2^ = 0.010). The interaction effect of the independent factors was also not significant (*F*(3, 228) = 0.673, *p* = 0.570, η_p_^2^ = 0.009). Dunnett’s C *post-hoc* tests demonstrated that once again, the mean scores in the HH cluster were significantly higher than the mean scores in the other three clusters. The mean scores in the ML cluster were also significantly lower than those in the LE and EE clusters (**Table 1** and **Figure 1B**).

**Figure 2:**
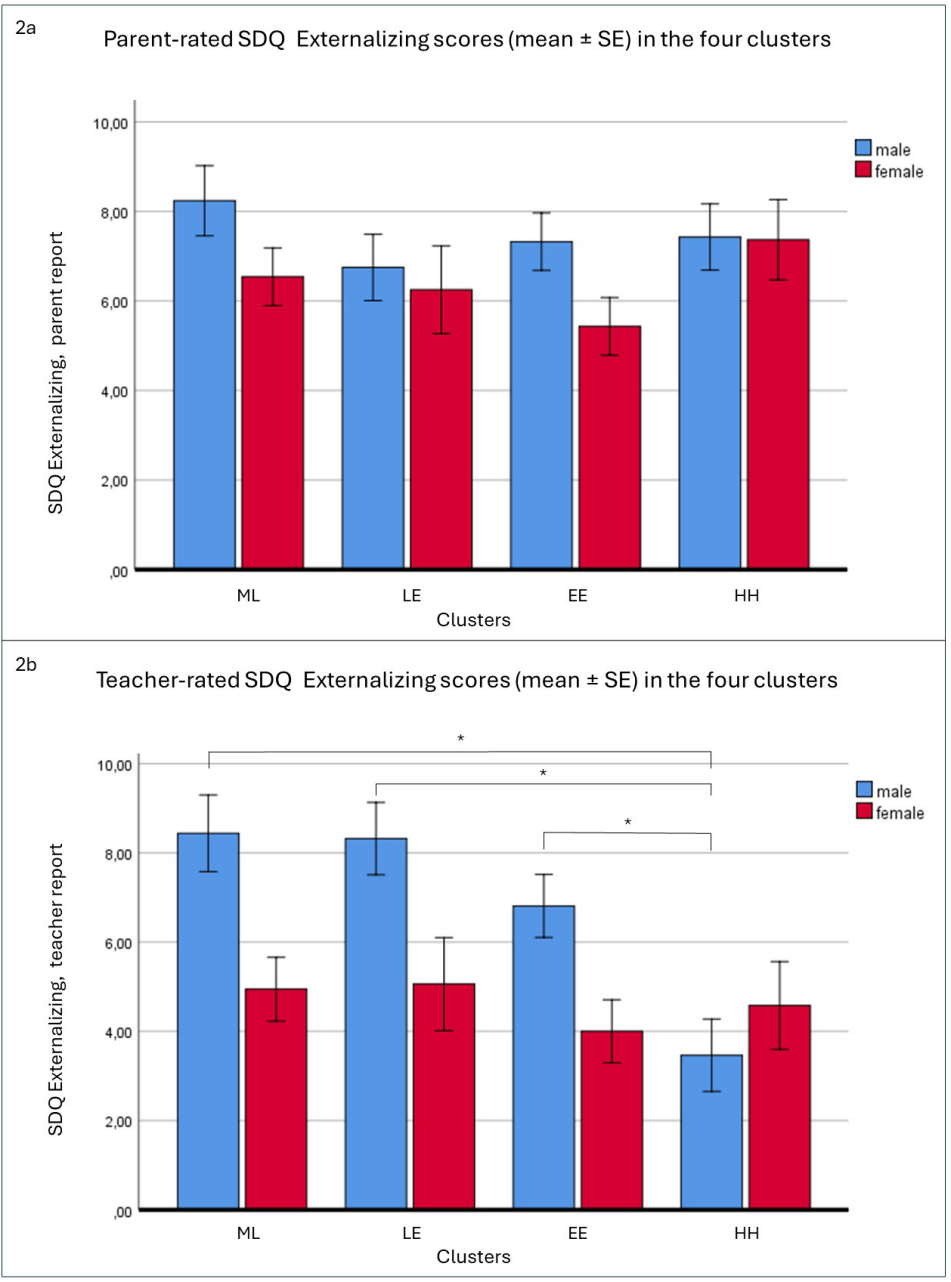
The effect of cluster membership and gender on parent **(A)** and teacher **(B)** rated SDQ Externalizing scores. N = 227. SDQ: Strengths and Difficulties Questionnaire. The labels of the four clusters according to the parent-report and teacher-report BIQ scales: HH, high-high; EE, elevated-elevated; ML, medium-low; LE, low-elevated; BIQ, Behavioral Inhibition Questionnaire; SE, standard error. * *p* < 0.05.

In the third model, with parent-rated SDQ Externalizing scores as the dependent variable, the model was not significant (*F*(7, 227) = 1.446, *p* = 0.188, η_p_^2^ = 0.044) (**Table 1** and **Figure 2A**).

Finally, when the teacher-rated SDQ Externalizing subscale was selected as the dependent variable, the ANOVA yielded a significant model (*F*(7, 227) = 5.606, *p* < 0.001, η_p_^2^ = 0.152). Both the main effect of cluster membership (*F*(3, 227) = 4.219, *p* = 0.006, η_p_^2^ = 0.055), and that of gender (*F*(1, 227) = 12.714, *p* <.001, η_p_^2^ = 0.055) were significant. Moreover, the interaction effect of cluster membership by gender was also significant (*F*(3, 227) = 3.029, *p* = 0.030, η_p_^2^ = 0.040). *Post-hoc* analysis revealed that, in boys, mean scores were lower in the HH cluster than in the other three clusters, whereas no differences were observed among the clusters in girls (**Table 1**, **Figure 2B**).

### 3.5 Parenting behaviors as predictors of cluster membership by gender

Multinomial logistic regression models were constructed to investigate the relationship between the MAPS subscales and membership in the four clusters, separately in the boys’ and girls’ samples. The HH cluster was selected as the reference category.

The goodness of fit tests indicated a good fit in both the boys’ sample (χ^2^(378) = 379.516, *p* = 0.468, Cox and Snell *R^2^* = 0.123, Nagelkerke *R^2^* = 0.131) and the girls’ sample (χ^2^(342) = 352.506, *p* = 0.336, Cox and Snell *R^2^* = 0.107, Nagelkerke *R^2^* = 0.115).

In the boys’ sample, the model was statistically significant (χ^2^(9, *N* = 164) = 21.518, *p* = 0.011) indicating that it was able to distinguish effectively between the clusters based on the predictor variables. However, the model was only marginally significant (χ^2^(9, *N* = 149) = 16.850, *p* = 0.051) in the girls’ sample.

In neither the girls’ nor the boys’ samples did any significant predictor emerge with regard to group membership of ML compared to HH.

In the comparison between LE and HH, hostility was a significant predictor in both samples. Each unit increase in MAPS-HS was associated with a slight decrease in the likelihood of membership in the LE cluster (**Table 2**). In boys, physical control was also a significant predictor. The probability of belonging to the LE cluster increased with the use of physical control, as opposed to the HH cluster.

**Table 2:** Results of the multinomial logistic regression analyses.

			*B*	*SE*	Wald	*df*	*p*	*OR* (95% *CI*)
boys (*N* = 164)	ML (ref = HH)	constant	-1.410	0.972	2.100	1	0.147	
		MAPS-HS	-0.099	0.460	0.046	1	0.829	0.906 (0.368 – 2.231)
		MAPS-LC	0.539	0.465	1.344	1	0.246	1.714 (0.689 – 4.262)
		MAPS-PC (ref = no use)	0.941	0.550	2.926	1	0.087	2.563 (0.872 – 7.538)
	LE (ref = HH)	Intercept	1.079	1.028	1.103	1	0.294	
		MAPS-HS	-1.070	0.506	4.470	1	0.034	0.343 (0.127 – 0.925)
		MAPS-LC	0.184	0.511	0.130	1	0.719	1.202 (0.441 – 3.275)
		MAPS-PC (ref = no use)	1.150	0.566	4.133	1	0.042	3.158 (1.042 – 9.572)
	EE (ref = HH)	Intercept	-0.107	0.888	0.014	1	0.904	
		MAPS-HS	-0.973	0.441	4.880	1	0.027	0.378 (0.159 – 0.896)
		MAPS-LC	1.110	0.433	6.568	1	0.010	3.033 (1.298 – 7.087)
		MAPS-PC (ref = no use)	0.705	0.495	2.025	1	0.155	2.023 (0.767 – 5.339)
girls (*N* = 149)	ML (ref = HH)	Intercept	0.742	1.152	0.416	1	0.520	
		MAPS-HS	-0.572	0.454	1.581	1	0.209	0.565 (0.232 – 1.376)
		MAPS-LC	0.538	0.462	1.357	1	0.244	1.712 (0.693 – 4.234)
		MAPS-PC (ref = no use)	0.298	0.523	0.326	1	0.568	1.348 (0.484 – 3.757)
	LE (ref = HH)	Intercept	2.529	1.339	3.568	1	0.059	
		MAPS-HS	-1.167	0.551	4.492	1	0.034	0.311 (0.106 – 0.916)
		MAPS-LC	0.114	0.566	0.040	1	0.841	1.121 (0.370 – 3.397)
		MAPS-PC (ref = no use)	0.373	0.611	0.373	1	0.541	0.689 (0.208 – 2.279)
	EE (ref = HH)	Intercept	1.934	1.160	2.778	1	0.096	
		MAPS-HS	-1.369	0.488	7.855	1	0.005	0.254 (0.098 – 0.663)
		MAPS-LC	0.805	0.475	2.870	1	0.090	2.236 (0.881 – 5.673)
		MAPS-PC (ref = no use)	0.362	0.534	0.459	1	0.498	1.436 (0.504 – 4.089)

MAPS-LC, Multidimensional Assessment of Parenting Scale; Lax Control subscale. MAPS-HS, Multidimensional Assessment of Parenting Scale; Hostility subscale. MAPS-PC, Multidimensional Assessment of Parenting Scale; Physical Control subscale (dummy-coded, 0 = no use; 1 = else). The labels of the four clusters according to the parent-report and teacher-report BIQ scales; respectively, HH, high-high; EE, elevated-elevated; LE, low-elevated; ML, medium-low. B, the unstandardized regression coefficient. SE, standard error. OR, odds ratio. 95% CI, 95% confidence interval of the OR.

For group membership of EE compared to HH, hostility was found to be a significant predictor in both genders. Each unit increase in MAPS-HS was associated with a slight decrease in the likelihood of membership in the EE cluster (**Table 2**).

However, in boys, lax control was also found to be significant. Each unit increase in MAPS-LC was associated with an increase in the likelihood of membership in the EE cluster, compared to the HH cluster (**Table 2**).

## 4 Discussion

Recent research has demonstrated that informant discrepancies do not simply result from measurement error. Rather, they may contain domain-specific information that contributes to our understanding of the phenomenon under investigation and informs the assessment and treatment planning (38, 39, 44–46). The necessity to utilize parent and teacher reports of children’s behavioral inhibition as a complementary rather than equivalent source of data has also been identified (12). However, no research has yet addressed this topic. Therefore, this study employed a cluster analytic approach to utilize both parent and teacher reports of behavioral inhibition in a sample of preschool children. The resulting clusters were characterized in terms of psychopathology and parenting behavior, with gender taken into account.

### 4.1 Parent and teacher report on behavioral inhibition is moderately correlated

The results indicated a positive association between parent and teacher reports, with an effect size that could be considered medium. This finding is consistent with previous studies (12, 32) and highlights the need for an explanation of informant discrepancies.

### 4.2 Gender differences in parent and teacher report

Despite the absence of gender differences in the parent report, the teacher report indicated that the mean scores of boys were significantly higher than those of girls. It has been posited that behavioral inhibition may be considered more gender-appropriate in girls than in boys (27, 59). Consequently, teachers may hold different expectations of girls’ and boys’ behavior.

### 4.3 Four clusters were identified according to the parent and teacher report

The two-step cluster analysis revealed a four-cluster solution. The four clusters were labelled as medium-low (ML), low-elevated (LE), elevated-elevated (EE), and high-high (HH) based on the parent and teacher report BIQ levels, respectively, and exhibited significant differences in parent and teacher report BIQ.

In the HH cluster, both parents and teachers reported similarly high levels of behavioral inhibition. The similarity of the teacher and parent scores provides greater confidence in inferring an intrinsic cause for the child’s behavior (45). This suggests that behavioral inhibition may be assumed to underlie the behavior in this group, as a temperamental trait. This is supported by the fact that twenty per cent of the sample fell into this cluster, a percentage that is consistent with the 15-20% of children with behavioral inhibition that has been identified in previous research (52). The cluster comprises 60% of the children who scored 132 or above on the parental report, which is the typical threshold for BIQ (51). Both the teachers and the parents indicated that the children exhibited significantly higher levels of internalizing problems than those observed in the other three clusters. This result can be explained by previous findings which indicated that behavioral inhibition is an early risk factor for internalizing disorders (8, 11). However, the cross-sectional design of this study also allows for the possible explanation that the higher level of behavioral inhibition may be a symptom of internalizing disorders or caused by a shared underlying factor (11). In contrast, according to the teacher’s report, the externalizing problem level was found to be significantly lower in this cluster than in the other three clusters, but only in boys. In preschool children, boys tend to show more externalizing symptoms than girls (59). However, our findings suggest that this gender difference disappears in highly inhibited children.

In the EE cluster, parents and teachers also reported a comparable level of behavioral inhibition. Although the reported level of behavioral inhibition was significantly lower than in the HH group, the normal to high-level range of behavioral inhibition in both parental and teacher reports could be indicative of a tendency towards inhibition. This can be attributed to a normal inhibition tendency in preschool children towards the unfamiliar, or alternatively, to an underlying temperamental characteristic that is well compensated by the environmental circumstances, including parental behavior (4, 29, 52). These external circumstances could be the difference in parental behavior (29, 52). In comparison to the HH cluster, an increase in the level of parental hostility was found to reduce the likelihood of membership in the EE cluster in both boys and girls. Conversely, an increase in the level of parental lax control was found to increase the likelihood of membership in the EE cluster but only in boys. As previous studies have demonstrated parental overcontrol, criticism and intrusiveness may influence the stability and impact of behavioral inhibition (15, 20, 21). In this way, the primary distinction between the HH and EE clusters can be attributed to the environmental conditions. On the other hand, it may reflect the natural variation in the underlying temperamental trait across children.

In the LE cluster, parents rated significantly lower on behavioral inhibition than teachers. While in the ML cluster there is only one level difference between the parental and teacher-rated behavioral inhibition levels, in the LE cluster, parents provided two levels lower evaluations than teachers. Although it is typical for parents to report a lower level of behavioral inhibition than teachers (7, 12), the large discrepancy between parental and teacher evaluations in the LE cluster indicates the presence of an external factor influencing the behavioral inhibition experienced by the teacher (45). It sheds light on a probable pathological pattern, which is corroborated by the fact that, in boys, parental physical control is significantly higher in the HH cluster. Although the LE cluster exhibits a significantly lower level of internalizing problems compared to the HH cluster, it shows a significantly higher level than the ML cluster, similar to the EE cluster. Additionally, in boys, compared to the HH cluster, the parental physical control is significantly higher.

In the ML cluster parents rated higher on the behavioral inhibition scale than teachers. This small discrepancy could be explained by the perspective bias, according to the ABC Model (45). In the preschool setting teachers have a greater basis for comparison. Additionally, they may find different difficulties or only minor ones than parents and they may recall memories of the children’s behavior accordingly (45). Teachers reported significantly lower levels of internalizing problems in this cluster than in the other three.

One noteworthy finding is that teachers tend to perceive HH children as exhibiting reduced levels of externalizing symptoms, whereas parents do not differentiate this group from the others with regard to the SDQ externalizing subscale score. This discrepancy may be due to the contextual dissimilarity between the home and the school settings; therefore, it can be explained by the diverging operations of the OTM (39). For children with behavioral inhibitions, the preschool setting is less familiar (33), which may reduce the likelihood of externalizing behavior in this context. Conversely, in the most familiar home setting, this effect is less pronounced, allowing for less inhibited behavior.

Furthermore, in accordance with the ABC model (45), a teacher’s perception of a child as behaviorally inhibited may influence the retrieval of memories of externalizing behaviors. Teachers’ opinions regarding the clinical significance of the child’s issues can also influence response tendencies. These factors may influence the retrieval of memories at the individual level.

Another significant finding is that parents tend to be less attuned to gender differences in externalizing behaviors, whereas teachers are more likely to differentiate between boys and girls. It is reasonable to hypothesize that while teachers may hold explicit or implicit gendered beliefs and expectations (60), parents may focus more on their child’s individual characteristics and behavior. The ABC model (45) predicts that teachers’ gendered expectations would affect how their memories are evoked about externalizing behaviors, which is consistently reported as more prevalent in boys than girls at the preschool age (e.g (59)).

Our results suggest that parental hostility may be associated with the extremities of the teacher-reported behavioral inhibition continuum (low/high). This means that the impact of parental hostility may be more pronounced in the preschool context; on the other hand, parental hostility may influence the child’s behavior in interaction with other factors such as the child’s temperament.

Taken together, the results suggest that parental practices may interact with temperamental characteristics to influence the developmental trajectory of children. Moreover, these factors may manifest differently in boys and girls.

### 4.4 Clinical implications

Our findings underscore the significance of integrating parent and teacher reports on behavioral inhibition in clinical settings (12). This will enhance the precision of the assessment. Moreover, the identification of particular patterns of informant discrepancies can furnish domain-specific information that assists the clinician in formulating hypotheses regarding the causes and correlates of children’s inhibited behavior in different contexts. This will inform the assessment process and guide treatment planning. The ABC Model (45) and the Needs-to-Goals Model (46) may be beneficial in conceptualizing behavioral inhibition in a clinical setting.

Research on early interventions for behavioral inhibition has produced promising results (51, 61). Parental behavior is a modifiable factor, therefore, an early intervention targeting parenting may alter the developmental course of behavioral inhibition and decrease the risk of internalizing disorders (2, 12). The results of this study indicate that a key objective of these interventions should be to reduce parental hostility.

### 4.5 Limitations

The results should be interpreted in light of the limitations of the study. Although the gender distribution of children was balanced, the parent sample predominantly comprised mothers, which could potentially influence the results. Further research is required to include other caregivers. A further limitation of the study is that data on the demographic characteristics of teachers were not collected. Consequently, an analysis of this or other aspects of teacher characteristics was not feasible.

A self-report questionnaire was employed to assess parents’ perceptions of their parenting behaviors, which may be influenced by social desirability, insight, and the parents’ attitudes toward parenting and their awareness of cultural norms. For instance, the use of physical control may have been underreported in Hungary due to the country’s zero-tolerance policy on physical punishment (62). The results must be interpreted in light of this limitation and further studies are needed to employ alternative methods, such as behavioral observation.

We used the MAPS (49) which measures three distinct negative parental behaviors, hostility, laxness and physical control. However, we did not measure other important parental behaviors such as overprotectiveness and overly sensitivity which were also linked to behavioral inhibition in previous studies (14–19). Further research is needed in this field.

A limitation of this research is the exclusive use of BIQ total score, which may overlook variations in behavioral inhibition across social and non-social contexts that could be captured by the BIQ subscales.

Although the BIQ and the SDQ internalizing subscale assess two interrelated yet conceptually distinct constructs, two items of the SDQ are overlapping to the phenomenon of behavioral inhibition (i.e., nervous or clingy in new situations, easily loses confidence; many fears, easily scared). This may influence the relationships between the variables.

The cross-sectional nature of the study precludes the ability to draw causal conclusions. For instance, previous studies have indicated that the relationship between behavioral inhibition and parenting may be bidirectional (14, 15, 20, 22, 23). Longitudinal studies are necessary to investigate their interrelations over time.

In our study, a cluster analytic approach was employed to integrate parent and teacher reports. Nevertheless, we did not propose an algorithm for clinical use how to capture unique variance of the different informants. Further research is needed in this field.

## 5 Conclusion

The findings indicate that the integration of parent and teacher reports on behavioral inhibition may enhance the identification of highly inhibited children. Furthermore, informant discrepancies on behavioral inhibition may contain domain-specific information, that could be utilized to gain a more comprehensive understanding of the causes and correlates of children’s inhibited behavior across different contexts.

## Data Availability

The datasets presented in this study are available in the Open Science Framework (OSF) repository: https://osf.io/f5tym/?view_only=d64a5bab5fd94bcd8fb6d672aa802ea7.
